# Listener Modeling and Context-Aware Music Recommendation Based on Country Archetypes

**DOI:** 10.3389/frai.2020.508725

**Published:** 2021-02-02

**Authors:** Markus Schedl, Christine Bauer, Wolfgang Reisinger, Dominik Kowald, Elisabeth Lex

**Affiliations:** ^1^ Johannes Kepler University Linz, Linz, Austria; ^2^ Linz Institute of Technology, Linz, Austria; ^3^ Utrecht University, Utrecht, Netherlands; ^4^ Know-Center GmbH, Graz, Austria; ^5^ Graz University of Technology, Graz, Austria

**Keywords:** music, recommender system, culture, country, clustering, context, user modeling, music preferences

## Abstract

Music preferences are strongly shaped by the cultural and socio-economic background of the listener, which is reflected, to a considerable extent, in country-specific music listening profiles. Previous work has already identified several country-specific differences in the popularity distribution of music artists listened to. In particular, what constitutes the “music mainstream” strongly varies between countries. To complement and extend these results, the article at hand delivers the following major contributions: First, using state-of-the-art unsupervized learning techniques, we identify and thoroughly investigate (1) country profiles of music preferences on the fine-grained level of music tracks (in contrast to earlier work that relied on music preferences on the artist level) and (2) country archetypes that subsume countries sharing similar patterns of listening preferences. Second, we formulate four user models that leverage the user’s country information on music preferences. Among others, we propose a user modeling approach to describe a music listener as a vector of similarities over the identified country clusters or archetypes. Third, we propose a context-aware music recommendation system that leverages implicit user feedback, where context is defined via the four user models. More precisely, it is a multi-layer generative model based on a variational autoencoder, in which contextual features can influence recommendations through a gating mechanism. Fourth, we thoroughly evaluate the proposed recommendation system and user models on a real-world corpus of more than one billion listening records of users around the world (out of which we use 369 million in our experiments) and show its merits vis-à-vis state-of-the-art algorithms that do not exploit this type of context information.

## 1 Introduction

Recommendation systems (or recommender systems) have become an important means to help users find and discover various types of content and goods, including movies, videos, books, and food (Ricci et al., 2015). As such, they represent substantial business value. In the music industry, recommender systems—powered by machine learning and artificial intelligence—have radically changed the market; they have even become major drivers in this industry. Essentially, music recommender systems (MRS) shape today’s digital music distribution (Schedl et al., 2015) and have become vital tools for marketing music to a targeted audience, as evidenced by the success of recommender-systems-featuring music streaming services such as Spotify, Deezer, or Apple Music. While MRS operate in a multi-stakeholder environment including platform providers, artists, record companies, and music consumers/listeners (Bauer and Zangerle, 2019), it is most commonly the music consumers/listeners, who are considered the users of an MRS. In the paper at hand, we also take this perspective.

Traditionally, content-based filtering and collaborative filtering (CF)—or hybrid combinations thereof—have been the most common algorithms to create recommender systems (Ricci et al., 2015). The former assumes that users will like items similar to the ones they liked in the past, and therefore selects items to recommend according to some notion or metric of similarity in terms of item content (e.g., music style, timbre, or rhythm) between the user’s liked items and unseen items from the catalog. In contrast, CF assumes that a user will prefer items that are liked by other users with similar preferences. In this case, items to recommend are, for instance, found by comparing the target user’s consumption or rating profile to that of the other users, identifying the most similar other users, and recommending what they liked (user-based CF). Alternatively, users and items can be directly matched via similarities computed in a joint low-dimensional representation of users and items (i.e., model-based CF).

Enhancing the classical approaches CF and content-based filtering, in recent years, researchers started to leverage additional information—beyond users, items, and their interactions—to improve recommendations. Recommender systems that consider user characteristics or information describing a situation are typically referred to as *context-aware recommendation systems* (Adomavicius and Tuzhilin, 2015). Next to considering time and location as contextual side information, taking information derived from the user’s country into account has been demonstrated to improve recommendation quality; for instance, cultural and socio-economic characteristics of the user’s country (Zangerle et al., 2018), or the user taste’s proximity to their country-specific music mainstream (“mainstreaminess”) (Bauer and Schedl, 2019).

Against this background, we approach the task of context-aware music recommendation based on country information; in contrast to most previous works, we consider user country in our approach without using any external information about the country, such as cultural, economic, or societal information. The reason is that respective data sources about countries (e.g., Hofstede’s cultural dimensions,^1^ the Quality of Government measures,^2^ or the World Happiness Report^3^) provide information on the country level, which may not necessarily reflect the circumstances of individual users and, thus, can introduce problems in the recommendation process. For instance, cultural values or income may be very unequally distributed among a country’s population.

To avoid this, instead of using external information derived from the user’s country, we leverage purely the self-reported country information of the users as available in the system, and investigate how behavioral data about music listening can be used to (1) identify archetypal country clusters based on track listening preferences, (2) how users can be modeled using the results of (1), and (3) how the resulting user models can be integrated into a state-of-the-art deep learning-based music recommendation algorithm.

As in many other domains, nowadays, deep neural network architectures dominate research in music recommendation systems, due to their ability to automatically learn features from low-level audio signals and their superior performance (Schedl, 2019). This article is no exception. We propose a multi-layer generative model in which contextual features can influence recommendations through a gating mechanism.

In this context, we formulate the following research questions:

**RQ1:** To what extent can we identify and interpret groups of countries that constitute *music preference archetypes*, from behavioral traces of users’ music listening records?
**RQ2**: Which are effective ways to model the users’ geographic background as a contextual factor for music recommendation?
**RQ3**: How can we extend a state-of-the-art recommendation algorithm, based on variational autoencoders, to include user context information, in particular, the geo-aware user models developed to answer RQ2?

In the remainder of this article, we first explain the conceptual foundation of our work and discuss it in the context of related research (Section 2). Subsequently, we detail the methods we adopt to investigate the research questions; in particular, we specify the approaches used for data preparation, clustering, user modeling, and track recommendation (Section 3). The results of our experiments on uncovering geographic music listening archetypes and on music track recommendation, altogether with a detailed discussion thereof, are presented in Section 4. Finally, Section 5 concludes the article with a brief summary of the major findings, a discussion of limitations, and pointers to future work.

## 2 Conceptual Background and Related Work

A multitude of factors have been found to influence an individual’s music preferences. There is a long history of research investigating the relationships between music preferences and, for instance, demographics (Colley, 2008; Bonneville-Roussy et al., 2013; Cheng et al., 2017), personality traits (Rentfrow and Gosling, 2003; Schäfer and Mehlhorn, 2017), and social influences (ter Bogt et al., 2011; Bonneville-Roussy and Rust, 2018).

In the middle of the nineteenth century emerged a cultural hierarchy in America (DiMaggio, 1982; Levine, 1988) where a high social status patronized the fine arts (referred to as “highbrow”) while all other forms of popular culture were associated with a lower status (referred to as ”middlebrow” or “lowbrow”). In the 1990s, a series of studies (Peterson and Simkus, 1992; Peterson and Kern, 1996) have defended the view that, for the elite, highbrow was being replaced by a consumption pattern termed “omnivorousness”. Cultural omnivorousness reflects that people’s taste includes both elite and popular genres. This was subsequently shown to hold for various countries (e.g., Holbrook et al., 2002; Coulangeon, 2003; Fisher and Preece, 2003). Also, the consumption practices of low status taste were reconceptualized: The earlier view that the lowbrow group would be willing to consume any entertainment on offer (Horkheimer and Adorno, 1972) was replaced by the finding that low status people tend to choose one form of entertainment and avoid others (Bryson, 1997). Thus, overall the view evolved from highbrow–lowbrow to omnivore–univore. Analyzing music consumption across eight European countries, Coulangeon and Roharik (2005) supported the “omnivore–univore” scheme rather than the former “highbrow–lowbrow” model. The omnivorous cultural taste was later found unstable over time (Rossman and Peterson, 2015), though. Katz-Gerro (2002) has shown that the dividing line of class distinctions varies across countries and also the genre associations to social classes deviate. She concludes that, while class matters, the main determinants of cultural preferences relate to gender, education, and age (Katz-Gerro, 1999). Coulangeon (2005) questions the earlier view on the reasons for the different tastes of higher- and lower-status classes: He challenges that it would be the upper class’ familiarity with the so-called “legitimate” culture and the little accessibility to that culture for the lower-status classes, that distinguished what the upper class and lower-status classes prefer. Instead, he attributes it to the diversity of the stated preferences of people of the upper class, whereas the preferences of members of lower-status classes appear more exclusive. Later work, studying music taste in the “modern age” (Nuccio et al., 2018), found little evidence that musical taste is indeed aligned with class position.

Although there is a multitude of factors that influence an individual’s music preferences that lead to a diversity of music created and listened to, there are (market) structures and other mechanisms that effect certain tendencies in what music is preferred within a particular community. For instance, the music recording industry is typically considered a globally oriented market (Dolata, 2013). Yet, studies have revealed the existence of national boundaries (Bauer and Schedl, 2018). There are various country-specific mechanisms that affect an individual’s music preferences and consumption behavior: Preferences are culturally shaped (Baek, 2015; Budzinski and Pannicke, 2017); music perceptions vary across cultures, for instance, with respect to mood (Morrison and Demorest, 2009; Stevens, 2012; Lee and Hu, 2014; Singhi and Brown, 2014); and countries have substantially different national market structures with respect to, for instance, available music repertoire due to copyright and licensing, advertising campaigns, local radio airplay, or quotas for national artists (Hracs et al., 2017; Gomez Herrera and Martens, 2018).

Knowledge about country-specific differences in music preferences can be explicitly used to improve music recommender systems, for instance, by leveraging information about the users’ geographic or cultural background. For instance, Vigliensoni and Fujinaga (2016) use a factorization machine approach for matrix factorization and singular value decomposition to integrate—amongst others—a user’s country as context information. Bauer and Schedl (2019) use a contextual pre-filtering approach (Adomavicius and Tuzhilin, 2015), where the user base is first segmented by user country, and a target person is then compared to other people from the very same country (in contrast to a comparison with the entire user base). Sánchez-Moreno et al. (2016) use a k-nearest neighbor (k-NN) approach integrating, among others, the user’s country as attribute. Zangerle et al. (2018) leverage further country-specific data sources; for each country, they use the respective scores on the cultural dimensions by Hofstede et al. (2005) as well as the scores of the World Happiness Report (Helliwell et al., 2016) to tailor recommendations to the individual.

The work at hand differentiates from related work in several aspects.

- First, although music preferences vary across countries, several studies (e.g., Moore et al., 2014; Pichl et al., 2017; Schedl et al., 2017; Bauer and Schedl, 2019) have shown similarities in music preferences between countries, typically identified with clustering approaches. Yet, to the best of our knowledge, the work at hand is the first one to integrate information on country similarities into the music recommendation approach.
- Second, while other work, most notably, Zangerle et al. (2018), reaches out to include external data about countries (such as economic factors, happiness index, cultural dimensions), the approach at hand remains independent from any external data sources, enabling platform providers to build a self-sustaining recommendation system. Such a system can rely exclusively on data that is contained in the provider’s platform, including users’ self-disclosed country information.
- Third, most existing research on music preferences and recommender systems considers music preferences on a genre level (e.g., Skowron et al., 2017; Adiyansjah et al., 2019) or artist level (e.g., Sánchez-Moreno et al., 2016; Bauer and Schedl, 2019). Research on country-aware music recommendation systems that provide recommendations on the track level is rare (e.g., Zangerle et al., 2018). However, the genre and the artist level may be too coarse-grained to reflect users’ music preferences, for several reasons. Music genres are vaguely defined (Beer, 2013; Sonnett, 2016; Vlegels and Lievens, 2017) and users’ perceptions thereof differ tremendously (van Venrooij, 2009; Brisson and Bianchi, 2019). Artists frequently cover several music styles throughout their career, where some tracks may be more favored than others for reasons including lyrics quality, the influence of associated music videos, over-exposure, or associations with unpleasant personal experiences (Cunningham et al., 2005). Accordingly, the work at hand investigates music recommendations on the track level to reflect users’ preferences in a more fine-grained manner than genre labels attributed to an artist’s overall repertoire could do.
- Fourth, while deep learning approaches are increasingly used for recommender systems in general and for music recommendation in particular, the integration of geographic aspects—especially user country—with deep learning for music recommendation is a particular asset of the work at hand. For instance, a recent survey on deep learning-based recommender systems (Batmaz et al., 2019) reports that extant research mainly uses textual information to capture context in approaches to context-aware recommender systems. The authors particularly consider context that is extracted from items (e.g., text documents) instead of users.

## 3 Methods

In the following, we detail how we gather and process the dataset used in our study, which contains information about users’ music listening behavior (Section 3.1). We then describe our approach to identify country clusters based on this dataset (Section 3.2). Finally, we elaborate on our approaches to create user models incorporating country information and we detail our neural network architecture that integrates these models (Section 3.3).

### 3.1 Data Acquisition and Processing

**FIGURE 1:**
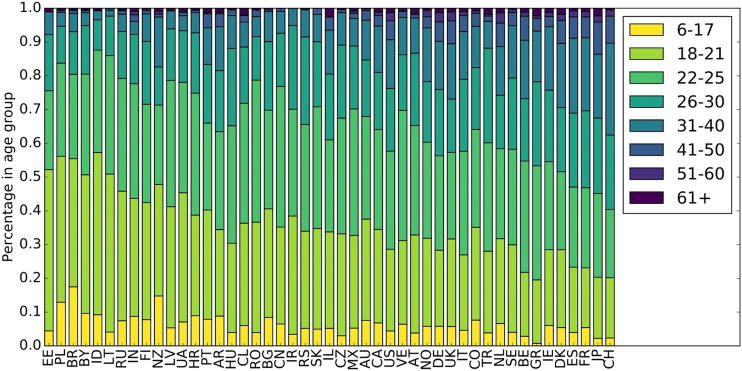
Distribution of age over countries. Countries are sorted in decreasing order of number of users from left to right.

**FIGURE 2:**
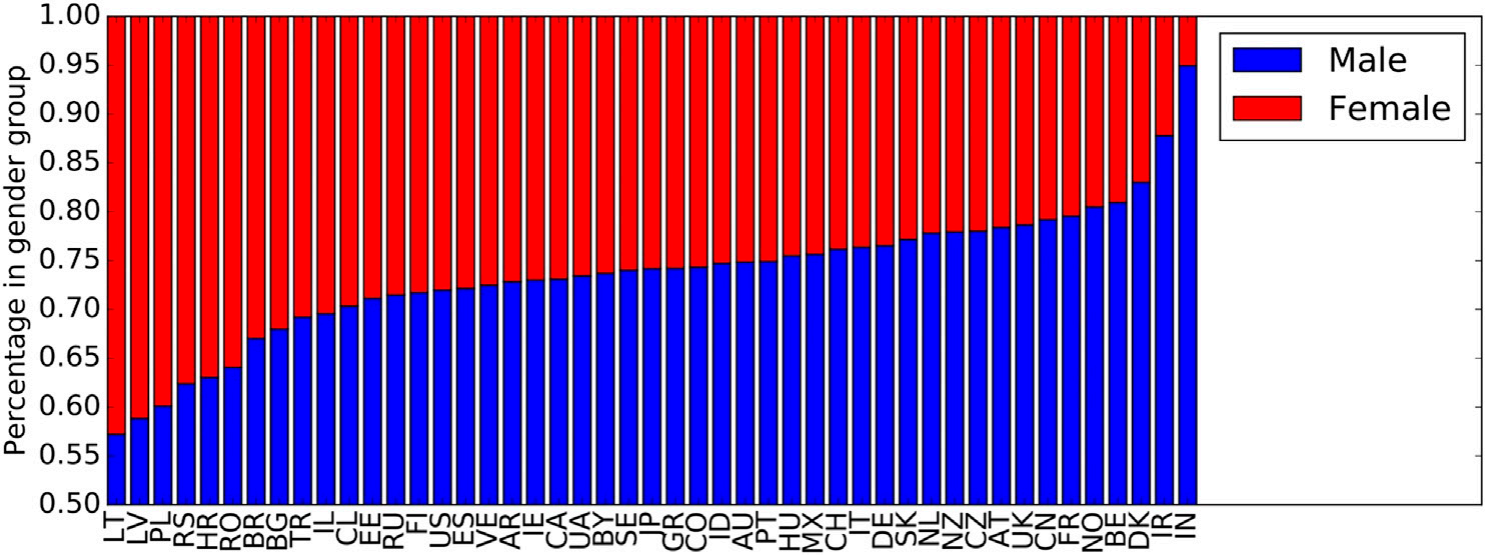
Distribution of gender over countries. Countries are sorted in decreasing order of number of users from left to right.

We base our investigations on the LFM-1b dataset (Schedl, 2016), which we filter according to our requirements as detailed below. The LFM-1b dataset^4^ contains music listening information for 120,322 Last.fm users, totaling to 1,088,161,692 individual listening events (LEs) generated between January 2005 and August 2014; the majority of LEs was created during years 2012–2014.^5^ Each LE is characterized as a quintuple of user-id, artist-id, album-id, track-id, and timestamp. The average number of LEs per user in the dataset is 8,879 (std. 15,962). For some users, also demographic data (country, age, and gender) is available in LFM-1b. More precisely, 46% of the users do provide information about their country, the same percentage do provide information about their gender, and 62% about their age. The majority of users who provide their country are from the United States (18.5%), followed by Russia (9.1%), Germany (8.3%), the United Kingdom (8.3%), Poland (8.0%), Brazil (7.0%), and Finland (2.6%). The mean age of the users who reveal it is 25.4 years (std. 9.4); the median age is 23 years. The age distribution differs significantly between countries, though. In Figure 1, we show the age distribution for the countries with at least 100 users (47 countries), categorized into age groups. The youngest users are found in Estonia and Poland, while the oldest users are Swiss and Japanese. Among the users who indicate their gender, 72% are male and 28% are female. These percentages differ, however, considerably between countries. In Figure 2, we therefore depict the ratios between genders, again for the top 47 countries in terms of number of users. While the Baltic countries Lithuania and Latvia have an almost equal share of male and female users, India and Iran show a very unequal distribution (around 90% male users).

**FIGURE 3:**
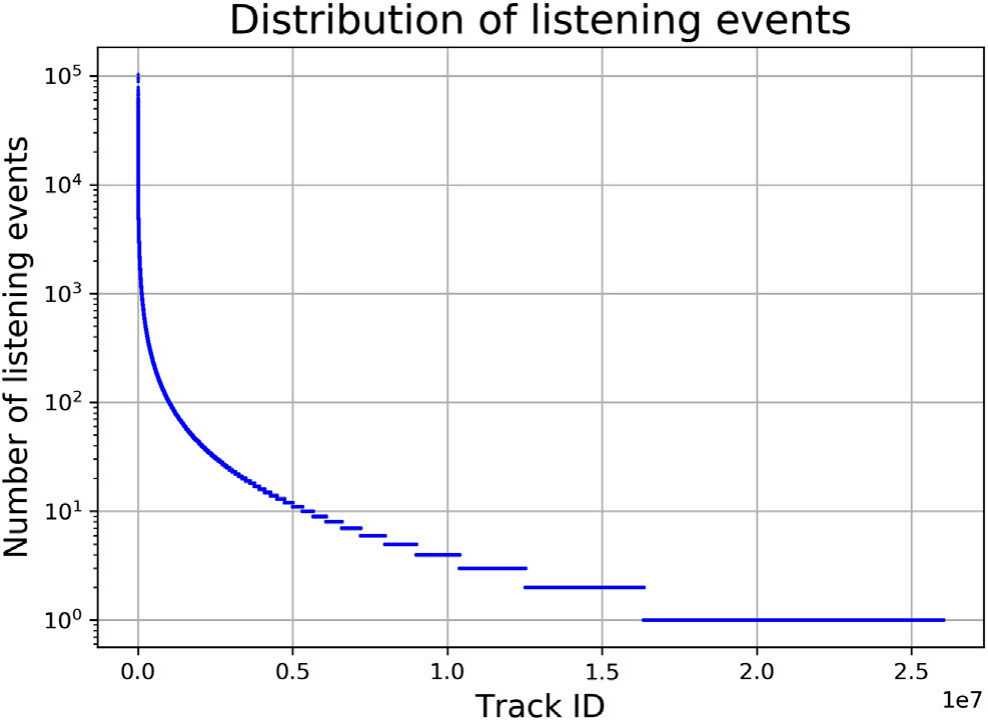
Distribution of number of listening events over all tracks (semi-log-scaled). Track identifiers are ordered by number of LEs.

As reported above, about 46% of users in the LFM-1b dataset disclose their country. For our country-specific analysis, we therefore only consider users (and their LEs) for whom country information is available. This results in a dataset of 55,186 users, who have listened to a total of 26,021,362 unique tracks. The distribution of the number of LEs over tracks is visualized in Figure 3.

**FIGURE 4:**
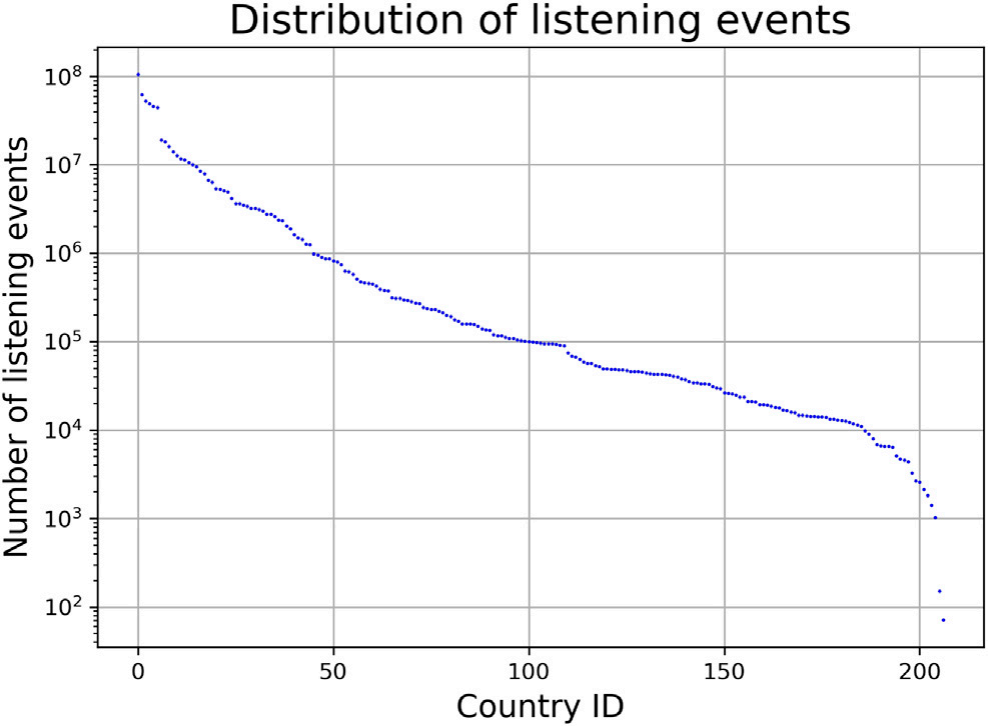
Distribution of number of listening events over all countries (semi-log-scaled). Country identifiers are ordered by number of LEs.

**FIGURE 5:**
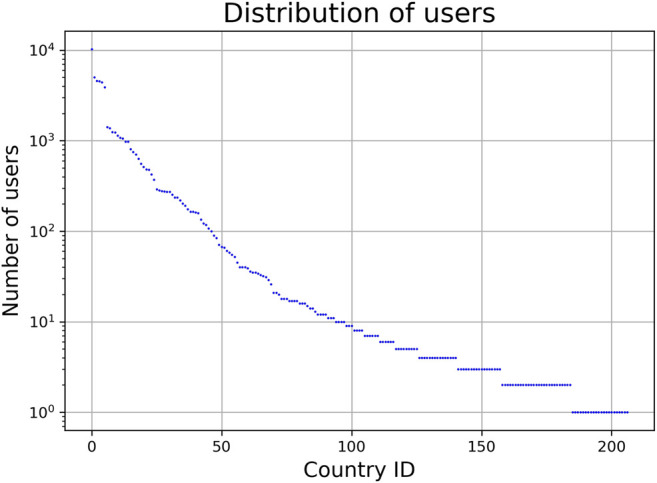
Distribution of number of users over all countries (semi-log-scaled). Country id ordered by number of users.

We subsequently reduce the data to decrease noise originating from the user-generated nature of the metadata in the LFM-1b dataset (in particular, misspellings and ambiguities), i.e., we filter out tracks and countries. This noise would otherwise likely cause distortions in future steps of our approach. First, we drop tracks that have been listened to less than 1,000 times, globally, resulting in a total of 122,442 tracks to consider further. Second, to minimize possible distortions caused by countries with a low number of LEs or a low number of unique users, we only consider countries with at least 80,000 LEs and at least 25 users. We chose these values as thresholds based on an empirical investigation of the distributions of LEs and of users over countries (cf. Figures 4 and 5, respectively). The former shows a flat characteristic around country-id 100, followed by a clear gap between country-id 110 and 111 (which corresponds to 80,000 LEs). The latter reveals a sudden drop at country-id 70 (which corresponds to 25 users). Applying this country filtering eventually results in 70 unique countries and a total of 369,290,491 LEs, which represents only a small drop of 1.5% (in comparison to 374,770,382 LEs created by users of all countries in the dataset). After these preprocessing steps, each country is represented as a 122,442-dimensional feature vector containing the LEs over all tracks.

### 3.2 Identifying Country Clusters and Archetypes

To cluster countries according to their citizens’ listening behavior, it is important to first normalize the data of each country to avoid distortions caused by different country sizes. To this end, we normalize each country’s feature vector to sum up to one.^6^ We next apply truncated SVD/PCA (Halko et al., 2001), reducing the dimensionality of the feature vectors to 100, while still preserving 99.8% of the variance in the data.^7^ Taking these 100-dimensional feature vectors as an input to a t-distributed Stochastic Neighbor Embedding (t-SNE) (van der Maaten and Hinton, 2008) and subsequently using OPTICS (Ankerst et al., 1999) enables us to visualize the data and identify clusters of countries sharing similar music listening behaviors.

T-SNE is a visualization technique that embeds high-dimensional data in a low-dimensional (typically, two-dimensional) visualization space, paying particular attention to preserving the local structure of the original data. It is particularly useful to disentangle data points that lie on more than one manifold. T-SNE represents proximities or affinities between pairs of data items by estimating the probability that the first data item will choose the second one as its nearest neighbor, and vice versa. In the original data space, this probability is modeled by means of a Gaussian distribution centered around each data item in the high-dimensional space; in the visualization space by means of a t-student distribution centered around each data item in the low-dimensional space. Kullback–Leibler divergence of the joint distributions between pairs of data points in the original space and in the visualization space is then minimized via gradient descent.

Ordering Points To Identify the Clustering Structure (OPTICS) is a density-based clustering method that creates a linear ordering of data items based on their spatial proximity. For this purpose, OPTICS first identifies core data points that have at least a certain number of neighbors in their vicinity (the minimum cluster size) and assigns a core distance to them, describing how dense the area around each core point is. Furthermore, a reachability distance between each pair of data items *o* and *p* is established, which is the maximum of (1) the distance between *o* and *p*, and (2) the core distance of *o*, whichever is bigger. Data items assigned to the same cluster have a lower reachability distance to their nearest neighbors than items that belong to different clusters. OPTICS subsequently creates an ordering of data items in terms of their reachability distance and identifies sudden changes in reachability between neighboring items, assuming that these correspond to cluster borders. The number of clusters is controlled by a parameter ξ that defines the minimum steepness (relative change in distance) between neighboring data items to be considered a cluster boundary.^8^

As for parameter optimization, we adopt a grid search strategy to identify a well-suited perplexity for t-SNE (5) and a minimum size of clusters, i.e., minimum number of data items in each cluster, for OPTICS (3).^9^ Please note that we use ISO 3166 2-digit country codes to refer to countries in this article.^10^

**FIGURE 6:**
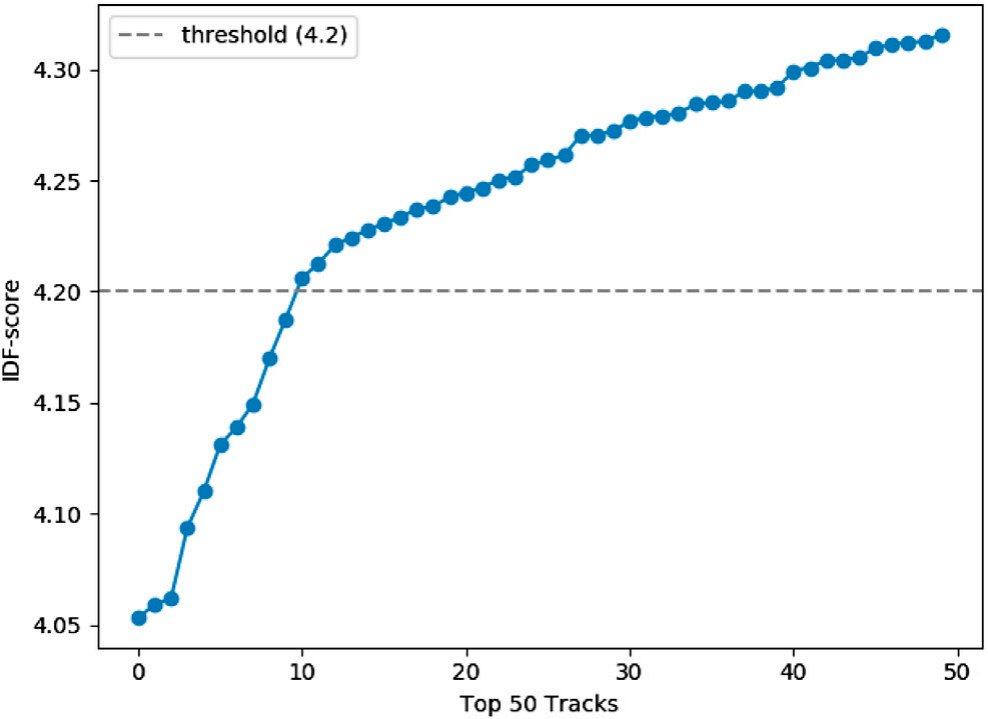
Inverse document frequency (IDF) scores for the top 50 tracks.

For an analysis of the identified clusters in a way that enables the establishment of archetypes of music preferences, we adopt the following approach. As shown in Figure 3, we observe a long-tail distribution of listening events over tracks, which means that a few dominating tracks are listened to by a lot of users, while most tracks are only listened to by a few users. Thus, these dominating tracks will also be popular among the list of top-tracks per cluster, which makes it hard to distinguish between the clusters and to interpret their corresponding archetypes. To overcome this, we adapt a scoring function similar to the inverse document frequency (IDF) (Jones, 1972) metric from the field of information retrieval, which assigns high scores to rarely occurring tracks and low scores to frequently occurring tracks. Formally, we define IDF for each track $t_{i}$ as $IDF\left(t_{i}\right)={\text{log}}_{10}\frac{N}{n_{i}}$, where *N* is the number of all listening events and $n_{i}$ is the number of LEs for track $t_{i}$. The distribution of IDF values of the top 50 tracks, in terms of $IDF\left(t_{i}\right)$, is plotted in Figure 6. In an empirical analysis, we identify 10 overall dominating tracks using a threshold of 4.2 on the IDF values (see Figure 6). These tracks are Rolling in the Deep by Adele, Somebody That I Used to Know by Gotye, Islands and Intro by The xx, Blue Jeans by Lana Del Rey, Supermassive Black Hole by Muse, Skinny Love by Bon Iver as well as Use Somebody, Sex on Fire and Close by Kings of Leon. We remove these tracks from further analyses when discussing archetypes as these are not suited to discriminate between clusters.

In our analysis of archetypes, we include genre annotations, which we obtain as follows. For all tracks in the dataset, we retrieve the top user-generated tags using the Last.fm API.^11^ Subsequently, we filter the tags of each track using a comprehensive list of music genres and styles from Spotify, called Spotify microgenres (Johnston, 2018). This list contains 3,034 genre names (as of May 2019 when we extracted them), including umbrella genres such as pop and country, as well as smaller niches such as Thai hip-hop, German metal, and discofox (Johnston, 2018). The fine-grained reflection of subtle differences in microgenres provides a more particularized basis for describing the clusters, compared to the use of a more coarse-grained taxonomy of music genres. We note as a limitation that the microgenre categories are defined in a similarly vague manner as coarse-grained taxonomies of music genre (Beer, 2013; Sonnett, 2016; Vlegels and Lievens, 2017); and the semantics associated with (micro)genre names have evolved over time so that a precise definition appears difficult. Relying on a big corpus of data where microgenres are visualized and sonified (see The Every Noise project^12^), we nevertheless believe that using the concept of microgenres helps future research to build upon our work. Further note that we rely on the top user-generated tags from the Last.fm community for attributing microgenres to tracks; the microgenre–track associations, thus, reflect the Last.fm community’s understanding of microgenres, which may not be congruent with the music experts’ understanding. Additionally, synonyms may be present in the user-generated tags and, thus, two different tags could be used interchangeably to annotate the same tracks (e.g., “Rap” and “HipHop”).

To allow interested readers to conduct further analyses of the identified clusters on a microgenre level, we release the full list of the top 20 tracks (and corresponding artists) per cluster, and we include—for each track and artist—all microgenre annotations.

### 3.3 User Modeling and Music Track Recommendation

We build our context-aware music recommendation approach on top of a variational autoencoder (VAE) model (Jordan et al., 1999). VAEs are a type of autoencoders (Kramer, 1991) that consist of an encoder, a decoder, and a loss function. In contrast to classic autoencoders, which learn encodings directly, VAEs learn the distribution of encodings using variational inference. Via sampling from the learned distribution, more representations of the same items can be generated given the same amount of training data. Thus, VAEs can learn more complex items than classic autoencoders.

We opted to extend the VAE architecture for collaborative filtering presented by Liang et al. (2018) because in a large-scale study conducted by Dacrema et al. (2019), the approach followed by Liang et al. (2018) was found the only deep neural network-based approach that outperformed equally well tuned non-deep-learning approaches. In addition, Liang et al. (2018) evaluated their VAE architecture on the Million Song Dataset (Bertin-Mahieux et al., 2011), a common benchmark in the music domain. They showed substantially superior performance compared to several baselines, in particular, the linear model weighted matrix factorization and collaborative denoizing autoencoders.

**FIGURE 7:**
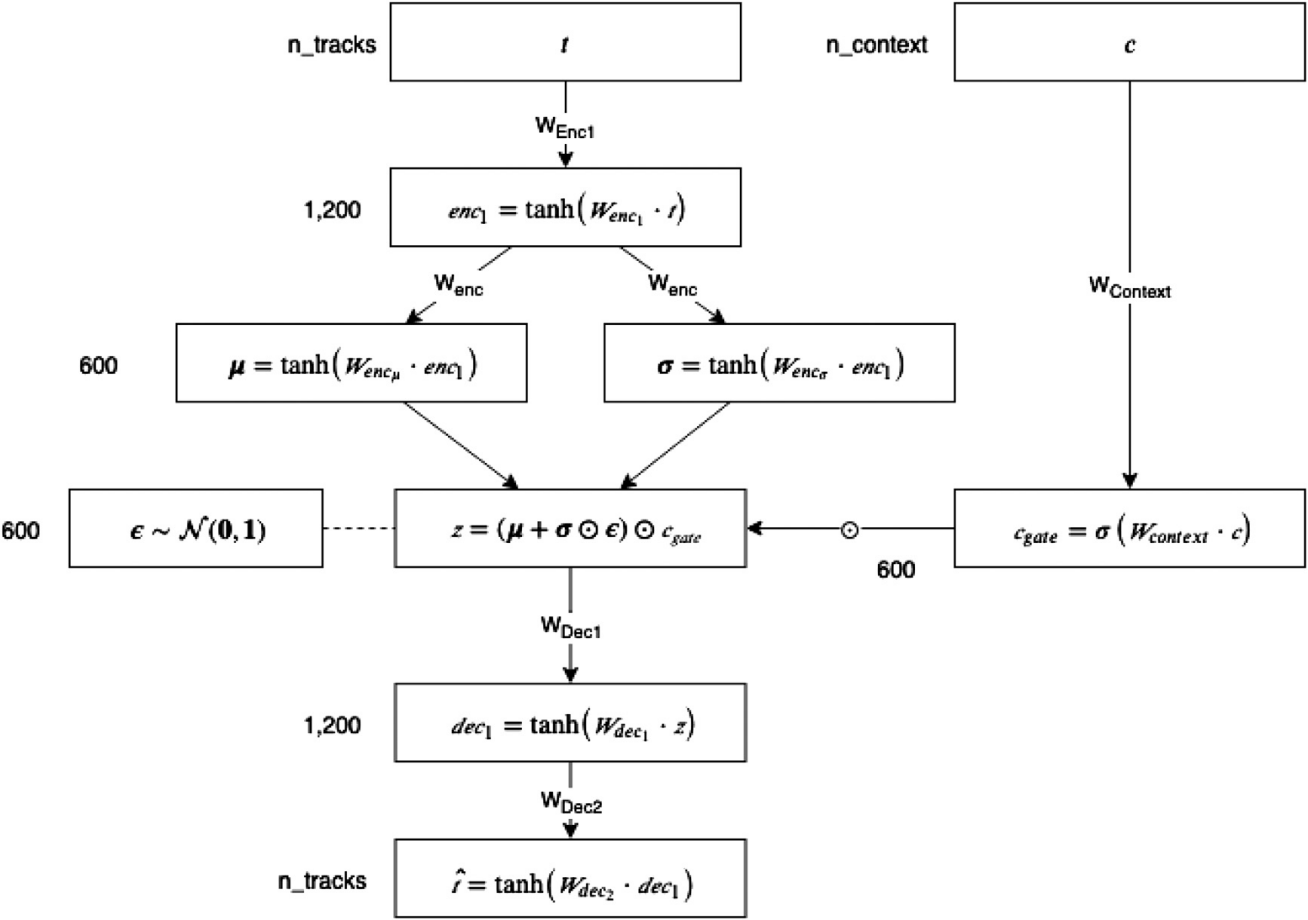
Architecture of the variational autoencoder with gated context information.

As depicted in Figure 7, we extend the VAE architecture by integrating context information using a gating mechanism. The gate output modulates the latent code in a way to incorporate context-based (country and cluster) differences of users. The abstract concepts are weighted based on how important the models deem them for a specific user group.^13^ Specifically, we model users in form of a 122,442-dimensional listening vector (i.e., n_tracks), which represents their track listening history, together with context information. We investigate four different ways to define a user’s context: (1) the user’s country, (2) the cluster membership of the user’s country, (3) the Euclidean distances between the user’s listening vector and all identified cluster centroids, and (4) the Euclidean distances between the user’s listening vector to all country centroids.

We derive context from the self-reported country of a user. For our VAE model with country context (i.e., model 1), a one-hot encoding of the 70 included countries is used, whereas for VAE with cluster context (i.e., model 2), context is determined by the user’s country membership in a cluster (see Table 1), resulting in a one-hot encoding of length 9. For the context models 3 and 4, we first calculate the cluster centroids, i.e., each track’s listening events of all users belonging to a cluster are summed and then normalized by the total amount of listening events across all tracks. Subsequently, for each user, the Euclidean distances between the respective user’s normalized feature vector and all cluster centroids are determined and used as context features for the VAE with cluster distances (i.e., model 3). Country distances are calculated accordingly, where each country is considered as its own cluster (i.e., model 4). Taken together, n_context is 70 in case of model 1 and model 4, and 9 in case of model 2 and model 3.

**TABLE 1:** Country clusters as determined by OPTICS with a minimum cluster size of 3, based on the output of a t-SNE visualization (perplexity of 5) on PCA-reduced country feature vectors (100 dimensions).

Cluster	Countries
0	ES, IT, IS, SI, PT
1	BE, NL, CH, SK, CZ, DE, AT, FI, PL
2	GB, EE, JP
3	AU, NZ, US, CA, PH
4	CL, CR, IL, UY
5	CO, MX, BG, GR
6	RO, EG, IR, TR, IN
7	BR, ID, VN, MY
8	LT, LV, UA, BY, RU, MD, KZ, GE
−1	AQ, FR, NO, ZA, IE, MK, AR, HR, RS, BA, HU, TW, DK, HK, SG, CN, KR, PE, TH, SE, PR, VE, GT

Countries identified as too noisy by OPTICS are represented as Cluster -1.

Our recommendation approach assumes that each user can be represented by a latent *k*-dimensional multivariate Gaussian, which is sampled, weighted by gates derived from context information, and transformed with a non-linear function to reconstruct the initial track listening history (cf. Figure 7). As mentioned before, our VAE model without contextual features is based on the work of Liang et al. (2018). To integrate context models, we extend the VAE by adding a gating mechanism to feed in contextual information according to the four ways detailed above. In a two-layer feed-forward neural network, the initial feature vector is encoded first into an intermediate representation enc1 and then into a latent *k*-dimensional multivariate Gaussian. The mean values μ and variance values σ are the outputs of the encoding network:

$$enc_{1}=\tanh\left(W_{enc_{1}}\cdot t\right)$$ (1)

$$\mu =\tanh\left(W_{enc_{\mu }}\cdot enc_{1}\right)$$ (2)

$$\sigma =\tanh\left(W_{enc_{\sigma }}\cdot enc_{1}\right)$$ (3)

We use tanh as a nonlinearity for all layers in the autoencoder. Based on our experiments (see Section 4.2.1), we set the size of $W_{enc_{1}}$ to n_tracks × 1,200 and both $W_{enc_{\mu }}$ and $W_{end_{\sigma }}$ to 1,200 × 600. This results in a length of 1,200 for $enc_{1}$ and 600 for the latent representation *z*. The user context, given by its input vector c is transformed by a dense layer with sigmoid nonlinearity into a context gate $c_{gate}$ of the same length as latent *z*. Next, the gate is applied with component-wize multiplication to *z*:

$$c_{gate}=\sigma \left(W_{context}\cdot c\right)$$ (4)

ε∼N(0,1) (5)

$$z=\left(\mu +\sigma ⊙\varepsilon \right)⊙c_{gate}$$ (6)

The weighted latent representation is then decoded back into the original space by a network with mirroring size but different learned parameters of the encoder:

$$dec_{1}=\tanh\left(W_{dec_{1}}\cdot z\right)$$ (7)

$$\hat{t}=\tanh\left(W_{dec_{2}}\cdot dec_{1}\right)$$ (8)

The detailed data flow and computation in each layer is visualized in Figure 7. Based on the known track history of a target user, the models generate a variational distribution $\hat{t}$. Top-*k* track recommendations are then retrieved by ranking the mean values of this distribution.

## 4 Results and Discussion

In the following, we present and interpret the results of our approach to identify country clusters and archetypes of music listening preferences (Section 4.1) and of the music track recommendation experiments (Section 4.2). We further connect the discussion to the initial research questions, which we answer in the context of the obtained results.

### 4.1 Clustering of Countries According to Music Listening Preferences

We present the identified clusters and discuss the relationship of the countries subsumed in each cluster beyond music preferences (Section 4.1.1), for instance, in terms of geographic proximity, linguistic similarities, and historical background. Furthermore, we discuss differences in user characteristics such as the users’ gender, age, and their listening patterns in terms of playcounts. In Section 4.1.2, we describe the characteristics of the clusters with respect to music preferences, i.e, we detail the track preferences that characterize the corresponding music archetypes.

#### 4.1.1 Identified Country Clusters

**FIGURE 8:**
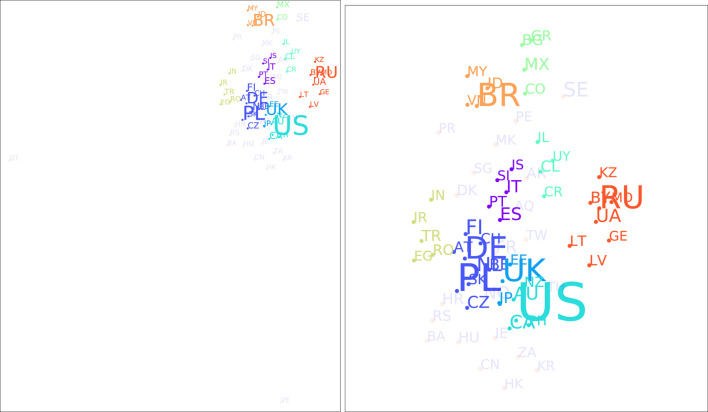
Results of t-SNE (perplexity of 5) and OPTICS (minimum cluster size of 3) on country feature vectors. The left part shows the full t-SNE output space, the right part a zoomed version onto the major clusters.

Using the approach described in Section 3.2, we can identify nine country clusters, which are presented in Table 1 and visualized in Figure 8. Cluster 0 contains Spain (ES), Portugal (PL), Italy (IT), Slovenia (SI), and Iceland (IS). Cluster 1 includes as many as nine countries: Belgium (BE), The Netherlands (NL), Austria (AT), Switzerland (CH), Germany (DE), Czech Republic (CZ), Slovakia (SK), Poland (PL), and Finland (FI). Cluster 2 refers to the United Kingdom (GB), Estonia (EE), and Japan (JP). Cluster 3 includes Australia (AU), New Zealand (NZ), the United States (US), Canada (CA), and the Philippines (PH). Cluster 4 refers to Chile (CL), Costa Rica (CR), Uruguay (UY), and Israel (IL). Cluster 5 contains Colombia (CO), Mexico (MX), Bulgaria (BG), and Greece (GR). Cluster 6 the following countries: Romania (RO), Egypt (EG), Iran (IR), Turkey (TR), and India (IN). Cluster 7 is composed of Brazil (BR), Indonesia (ID), Vietnam (VN), and Malaysia (MY). Cluster 8 encompasses eight countries: Lithuania (LT), Latvia (LV), Ukraine (UA), Belarus (BY), Russia (RU), Moldova (MD), Kazakhstan (KZ), and Georgia (GE).

Four of the countries in Cluster 0 are geographically tied together, sharing national borders (i.e., Spain (ES), Portugal (PL), Italy (IT), and Slovenia (SI)). Only Iceland (IS) is geographically dislocated. Furthermore, Spain (ES), Portugal (PL), and Italy (IT) share their roots in Romance language. Moreover, there is a Slovene minority in Italy (IT), which may lead to partly similar music preferences in Slovenia (SI) and Italy (IT).

**FIGURE 9:**
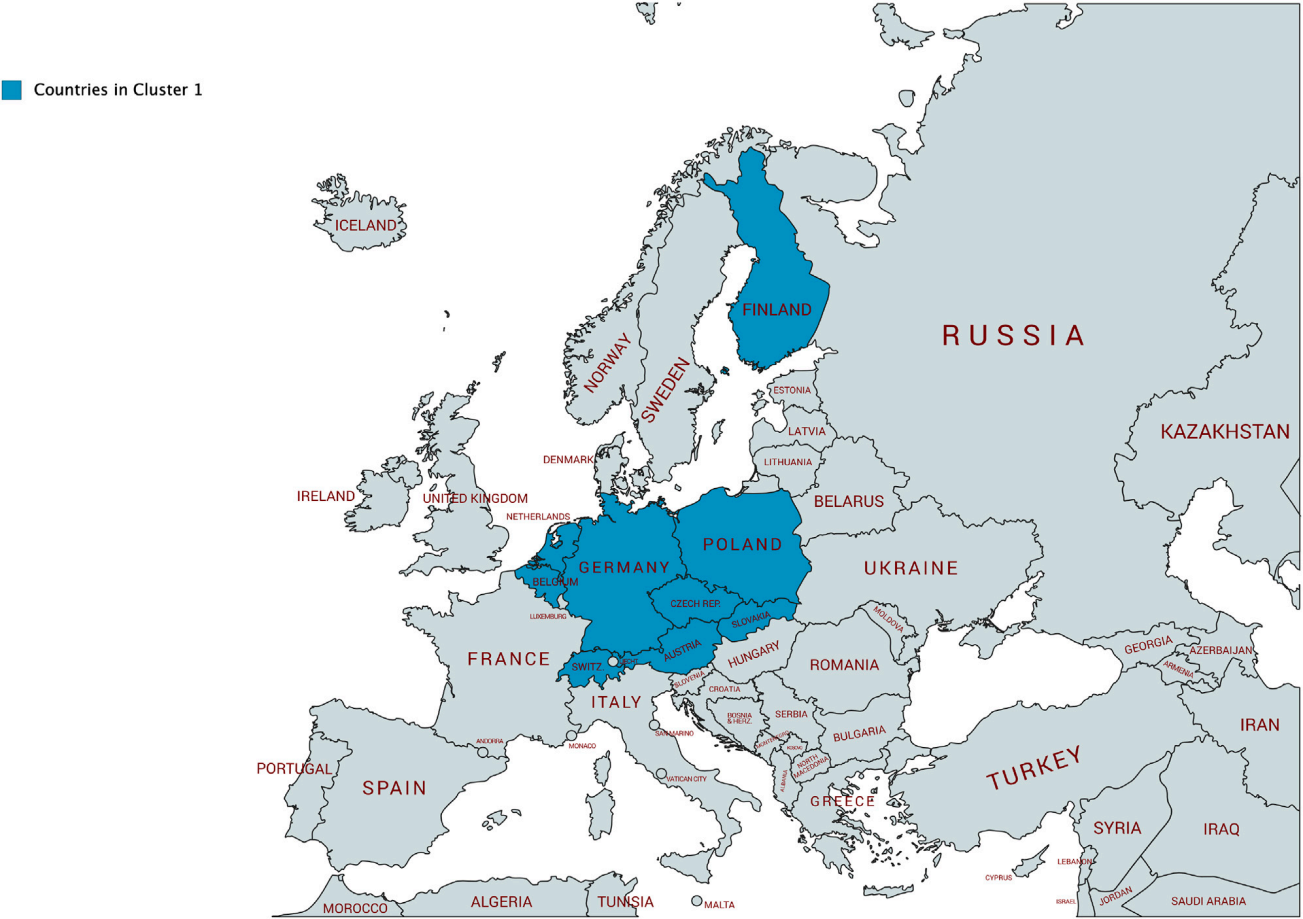
Countries in Cluster 1 on a map.

Cluster 1 contains nine countries. Belgium (BE) and the Netherlands (NL) are neighboring countries and share the official language spoken (note, Belgium (BE) has two official languages). Austria (AT), Switzerland (CH), and Germany (DE) share the German language (note, Switzerland (CH) has four official languages). Czech Republic (CZ) and Slovakia (SK) are not only neighboring countries, but actually formed one joint country until 1992. The languages spoken in the Czech Republic (CZ), Slovakia (SK), and Poland (PL)—a neighboring country to the former two—show strong linguistic similarities. Altogether, we can see that Belgium (BE), the Netherlands (NL), Austria (AT), Switzerland (CH), Germany (DE), Czech Republic (CZ), Slovakia (SK), and Poland (PL) are geographically connected, sharing national borders (cf. Figure 9). Only Finland (FI) is geographically disconnected from the other countries in this cluster.

**FIGURE 10:**
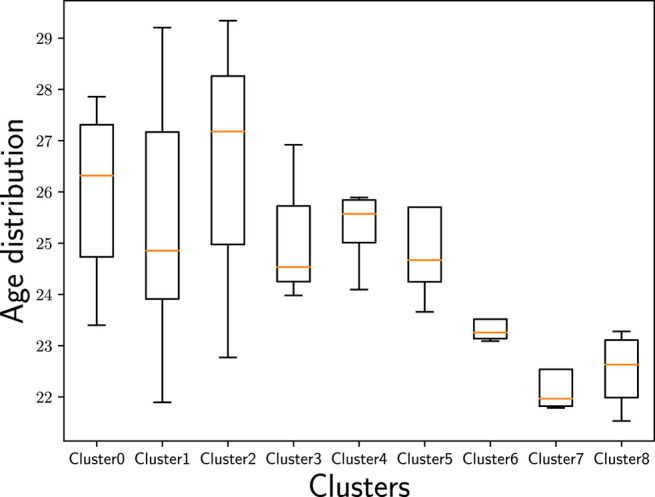
Age distribution of users in the identified country clusters. While the oldest users can be found in Cluster 2, the youngest can be found in Cluster 7.

**FIGURE 11:**
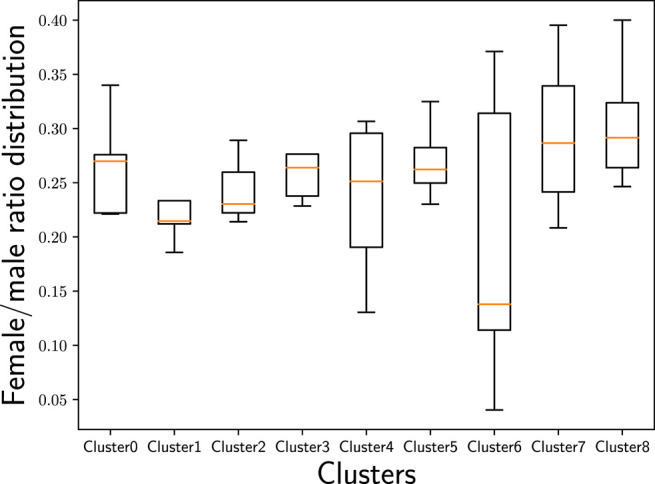
Distribution of users’ average playcount in the identified country clusters. While the highest average playcount can be found in Cluster 2, the lowest one can be found in Cluster 6.

Cluster 2 delivers a highly surprising result because it contains three countries that are geographically far away from each other without any linguistic similarities or close historical connections: the United Kingdom (GB), Estonia (EE), and Japan (JP). The United Kingdom (GB) and Estonia (EE) are located at the Northwest and the Northeast of Europe—thus, at the opposite borders of Europe; Japan (JP) is even almost 8,000 km farther east of Estonia (EE). Although this cluster contains only three countries, with Japan (JP) and the United Kingdom (GB), it embraces two of the largest music markets worldwide (Statista Research Department, 2019). Interestingly, the United Kingdom (GB) is not part of Cluster 3 that includes most English-speaking countries. Considering the age distribution (Figure 10) in the identified country clusters, we find that Cluster 2 shows the highest average age with a relatively large span.^14^ Furthermore, Cluster 2 shows by far the highest average playcount per user for the countries in this cluster (Figure 11). This indicates that users in this cluster are characterized as being ‘power listeners’. As the combination of countries in this cluster seems surprising, age and listening intensity may be the hidden—though determining—aspects for the emergence of this cluster.

The major connector of the countries in Cluster 3 is that they are all English-speaking countries: Australia (AU), New Zealand (NZ), United States (US), Canada (CA), and the Philippines (PH), where English is one of the two official languages in both Canada (CA) and the Philippines (PH).

Cluster 4 comprises the countries Chile (CL), Uruguay (UY), Costa Rica (CR), and Israel (IL). Both Chile (CL) and Uruguay (UY) are located in South America and are connected by their language: Spanish. The official language in Costa Rica (CR) is Spanish as well; located in Middle America, the geographic distance to Chile (CL) and Uruguay (UY) is not far. Israel (IL), in contrast, is a country in the Middle East and is, thus, geographically disconnected from the other three countries in this cluster.

Cluster 5 contains two Latin-American countries as well as two countries in Southeastern Europe. The Latin-American countries, i.e., Mexico (MX) and Colombia (CO), are both Spanish-speaking countries. With Mexico (MX) located in the Southern part of North America and Colombia (CO) being part of South America, these are no neighboring countries, though. The two countries in Southeastern Europe, i.e., Bulgaria (BG) and Greece (GR), share a border. Thus, the cluster contains two country groups, which are geographically spread.

**FIGURE 12:**
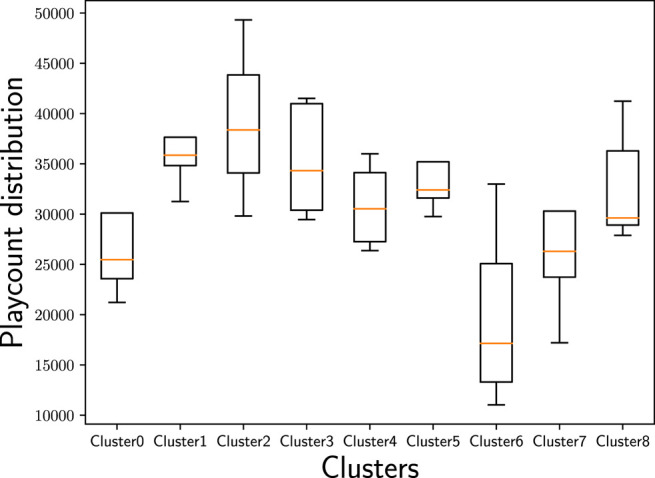
Female/male ratio distribution of users in the identified country clusters. We find that the female/male ratio is most unevenly distributed in Cluster 6 and most evenly distributed in Cluster 7.

The countries in Cluster 6 are geographically connected, centered around countries being part of the Middle East—Turkey (TR), Iran (IR), and Egypt (EG)—and flanked by Romania (RO), that has historical relations to the others due to the Ottoman Empire, and India (IN), that is adjacent to the Middle East and, thus, shows a geographical proximity to the other countries in this cluster. Furthermore, all the countries in Cluster 6 are very diverse when it comes to the various (minority) languages spoken, which may also be reflected in music preferences. Considering the female/male ratio of users (Figure 12) in the identified country clusters, we find that Cluster 6 shows the most unevenly distributed ratio across the countries in this cluster. Despite the wide span of female/male ratios in this cluster’s countries, Cluster 6 is the cluster with the overall lowest female/male ratio compared to the other clusters. With respect to age (Figure 10), this cluster comprises rather young users in our sample of the Last.fm community (with the average age of users in the Clusters 7 and 8 being even younger, though). Overall, with respect to age and gender, Cluster 6 seems to have a differentiating profile compared to the other clusters. Furthermore, Cluster 6 shows by far the lowest average playcount per user (Figure 11). This low number could be the result of a listening pattern that is shaped by cultural aspects, but could, for instance, also be the consequence of limited access to the resources (e.g., broadband Internet connection, streaming platforms operating in the respective countries, licenses for music content). Considering those and similar aspects is a fruitful path for future research.

Cluster 7 covers three neighboring countries (with maritime borders) in the Southeast of Asia—Indonesia (ID), Vietnam (VN), and Malaysia (MY)—and Brazil (BR) in South America. The three countries in the Southeast of Asia have many similarities, including common frames of reference in history, culture, and religion; also their national languages are closely related. From a geographic perspective, Brazil (BR) appears being disconnected from the other countries in this cluster. The connection of Brazil (BR) with Indonesia (ID) and Malaysia (MY) is that all three countries have formerly been Portuguese colonies (Bada, 2018). Whether this historical connection is indeed also conclusive for similar music preferences is subject to further research. Referring back to Figure 10, where we plot the age distribution for the identified country clusters, and Figure 12, where we plot the female/male ratio, we see that Cluster 7 shows the lowest average age and is close to the highest female/male ratio. Furthermore, the female/male ratio is very evenly distributed in Cluster 7. We, thus, suspect that age and gender are the hidden factors construing this cluster or, at least, accentuating it.

**FIGURE 13:**
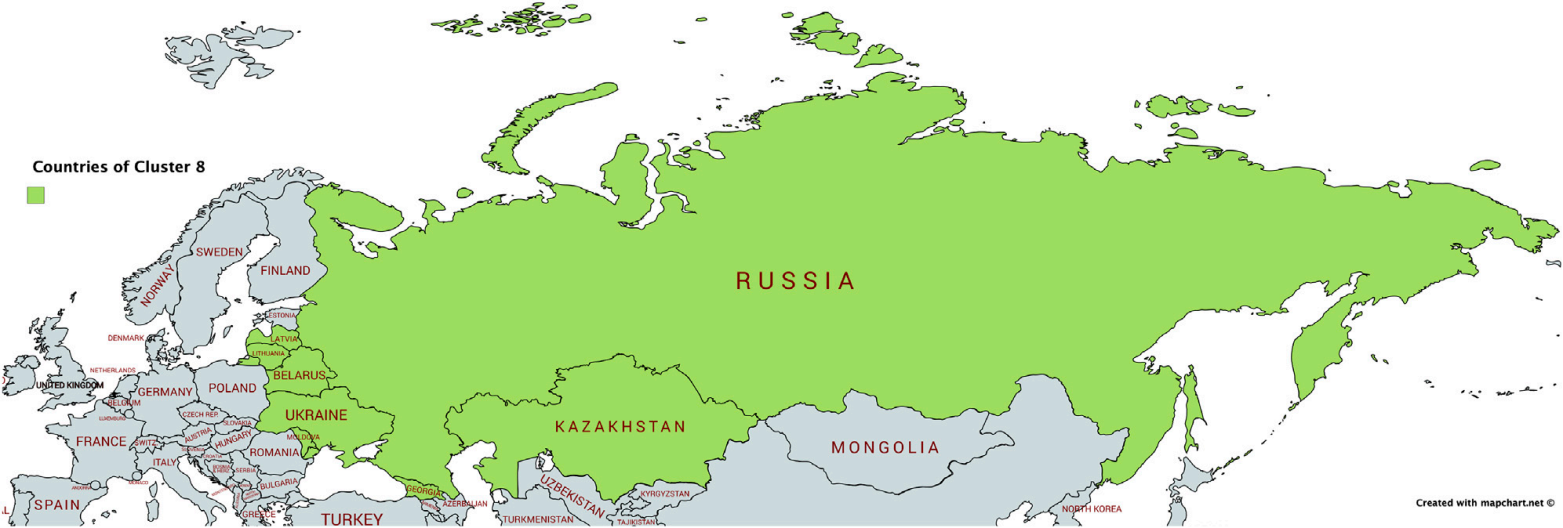
Countries in Cluster 8 on a map.

As can be seen from Figure 13, Cluster 8 comprises nine countries that are in geographical proximity: the Baltic countries Lithuania (LT) and Latvia (LV), the Russian Federation (RU), Ukraine (UA), Belarus (BY), Moldova (MD), Kazakhstan (KZ), and Georgia (GA). Besides being characterized by the geographic proximity, these countries share a history of having been part of the Russian empire. Russian is a major (or influential) language in all of the countries in this cluster (Central Intelligence Agency, 2019).

Overall, we note that the country clusters show different characteristics with respect to age (Figure 10), gender (Figure 12), and average playcount per user (Figure 11). With respect to age, we find especially large differences between the Clusters 2 and 7: While the highest average age can be found in Cluster 2, the lowest average age can be found in Cluster 7. The female/male ratio is high in Cluster 7 and also evenly distributed. In contrast, the female/male ratio is most unevenly distributed in Cluster 6 with a high span of ratios across the countries in this cluster; and overall, the ratio is—in comparison to the other clusters—very low. With respect to the average playcount per user, it is also the Clusters 2 and 6 that show the largest differences: Among the users in Cluster 2 there seems to be a high ratio of ‘power listeners’, whereas the average playcount of users in Cluster 6 is low in comparison. Overall, it can, thus, not be rejected that those and similar aspects may be hidden factors that accentuate the differentiation between the clusters or may even be indicative for the emergence of those clusters.

#### 4.1.2 Characteristics of the Identified Clusters and Music Preference Archetypes

To address the question what characterizes the various clusters in terms of music preferences, we use the approach described in Section 3.2 to identify the most important tracks and genres for each cluster. Table 2 provides a list of the 10 tracks with the highest playcounts per cluster (after the IDF-based filtering explained in Section 3.2) and their genre annotations;^15^ for genre annotations, we rely on the user-generated annotations retrieved from the Last.fm API and aligned with the Spotify microgenres, as described in Section 3.2. These most important tracks define the music preference archetypes corresponding to each cluster.

**TABLE 2:** The 10 most popular tracks per cluster. Playcount refers to the total number of listening events by the users in each cluster.

Cluster no.	Track title	Artist	Playcount within cluster	Track genres
0	Mr. Brightside	The Killers	4,248	rock, indie rock, alternative rock
	Uprizing	Muse	3,955	alternative rock, rock, progressive rock
	I Bet You look Good on the Dancefloor	Arctic Monkeys	3,835	indie rock, rock, alternative rock
	Fluorescent Adolescent	Arctic Monkeys	3,772	indie rock, rock, alternative rock
	VCR	The xx	3,597	electronic, indie rock, indie pop
	Reptilia	The Strokes	3,394	indie rock, rock, alternative rock
	Mardy Bum	Arctic Monkeys	3,345	indie rock, rock, alternative rock
	Hoppípolla	Sigur Rós	3,336	post-rock, ambient, post-rock
	There Is a light that Never Goes out	The Smiths	3,289	new wave, rock, brit-pop
	Teardrop	Massive Attack	3,260	triphop, electronic, downtempo
1	Set Fire to the Rain	Adele	20,460	soul, pop, singer/songwriter
	Little Lion Man	Mumford & Sons	20,160	folk, indie folk, banjo
	Otherside	Red hot Chili Peppers	19,469	rock, alternative rock, funk
	Radioactive	Imagine dragons	19,338	rock, indie rock, alternative rock
	VCR	The xx	19,220	electronic, indie rock, indie pop
	Heart Skipped a Beat	The xx	19,004	electronic, indie rock, rock
	Teardrop	Massive Attack	18,810	triphop, electronic, downtempo
	Sail	AWOLNATION	18,728	electronic, rock, indie rock
	The Pretender	Foo Fighters	18,636	rock, alternative rock, grunge
	Cosmic Love	Florence + the Machine	18,486	indie pop, rock, pop
2	There Is a Light That Never Goes Out	The Smiths	7,479	new wave, rock, brit-pop
	Mr. Brightside	The Killers	7,128	rock, indie rock, alternative rock
	Little Lion Man	Mumford & Sons	6,979	folk, indie folk, banjo
	R U Mine?	Arctic Monkeys	6,408	indie rock, rock, alternative rock
	I Bet You look Good on the Dancefloor	Arctic Monkeys	6,302	indie rock, rock, alternative rock
	I Miss You	Blink-182	6,295	rock, punk, pop-punk
	Teardrop	Massive Attack	6,187	triphop, electronic, downtempo
	The Cave	Mumford & Sons	6,150	folk, indie folk, banjo
	VCR	The xx	6,147	electronic, indie rock, indie pop
	Harder Better Faster Stronger	Daft Punk	6,083	electronic, house, electronica
3	It Ain’t Cool To Be CRAZY ABOUT YOU	George Strait	19,048	country, traditional country,
	Electric Feel	MGMT	18,108	electronic, electronica, indie pop
	Little Lion Man	Mumford & Sons	17,089	folk, indie folk, banjo
	Time to Pretend	MGMT	16,802	electronic, indietronica, electronica
	Flume	Bon Iver	16,032	folk, singer/songwriter, indie folk
	In the Aeroplane Over the Sea	Neutral Milk Hotel	15,753	indie rock, folk, lofi
	Midnight City	M83	15,635	electronic, electro-pop, electro
	1901	Phoenix	15,591	indie pop, electronic, indie rock
	Such Great Heights	The Postal Service	15,481	electronic, indie pop, electronica
	The Cave	Mumford & Sons	15,412	folk, indie folk, banjo
4	Mephisto	Dead Can Dance	2,468	ambient, medieval, folk
	3 Libras	A Perfect Circle	1,284	alternative rock, progressive rock, rock
	Ariane	Nova	1,238	–
	World’s End	Hatsune Miku & Megurine Luka	1,228	–
	Mr. Brightside	The Killers	1,109	rock, indie rock, alternative rock
	Las Fuerzas	Dënver	1,080	–
	Jeremy	Pearl Jam	1,069	Grunge, rock, alternative rock
	Reckoner	Radiohead	1,064	Alternative rock, rock, experimental
	Them Bones	Alice in Chains	1,057	Grunge, rock, alternative rock
	Nude	Radiohead	1,050	Alternative rock, rock, electronic
5	Häaden Two	Robert Fripp	11,616	–
	The Smile	Phase	7,898	alternative rock, progressive rock, art rock
	Ibidem	Phase	7,858	alternative rock, art rock, rock
	Perdition	Phase	7,752	rock, psychedelic rock, progressive rock
	Transcendence	Phase	7,690	psychedelic rock, rock, alternative rock
	Hypoxia	Phase	7,614	psychedelic rock, rock, alternative rock
	Static	Phase	6,988	rock, progressive rock, space rock
	A Void	Phase	6,913	rock, alternative rock, indie rock
	Static (live)	Phase	6,877	progressive rock, psychedelic rock, rock
	Evening On My Dark Hillside	Phase	6,793	psychedelic rock, rock, alternative rock
6	If I Could	Sophie Zelmani	13,420	singer/songwriter, pop, folk
	I Can’t Change [New Song]	Sophie Zelmani	13,409	–
	Without God	Katatonia	8,024	doom metal, metal, death metal
	Day	Katatonia	7,947	doom metal, metal, progressive metal
	Lady of the Summer Night	Omega	6,787	Rock
	Sorrow	Pink Floyd	6,485	progressive rock, rock, classic rock
	Equinoxe Part 5	Jean Michel Jarre	6,457	ambient, electronic rock,
	Gammapolis	Omega	5,958	classic rock, progressive rock, space rock
	To Know You	Sophie Zelmani	4,783	singer/songwriter, folk, pop
	To Know You (Alt. Version)	Sophie Zelmani	4,641	–
7	Set Fire to the Rain	Adele	17,247	soul, pop, singer/songwriter
	Fluorescent Adolescent	Arctic Monkeys	13,007	indie rock, rock, alternative rock
	Parade	Garbage	11,770	rock, alternative rock, pop
	National Anthem	Lana Del Rey	11,602	indie pop, pop, triphop
	Skyscraper	Demi Lovato	11,451	pop, pop-rock, disney
	Come & Get It	Selena Gomez	11,387	pop, electro-pop, dubstep
	Pumped Up Kicks	Foster the People	11,171	indie pop, pop, indie rock
	Dark Paradise	Lana Del Rey	11,056	pop, indie pop, chamber-pop
	Heart Attack	Demi lovato	10,606	pop, electro-pop, pop-rock
	You Only Live Once	The Strokes	10,501	indie rock, rock, alternative rock
8	Another Bottle Down	Asking Alexandria	19,779	post-hardcore, metal-core, screamo
	Only You	Savage	17,657	disco, pop, new wave
	…Meltdown	Enter Shikari	16,320	post-hardcore, trance-core, electronic
	What You want	Evanescence	12,345	rock, alternative metal, Gothic rock
	Gandhi Mate Gandhi	Enter Shikari	12,273	post-hardcore, electronic, dubstep
	Dexter	Ricardo Villalobos	11,889	minimal, minimal techno, electronic
	Paradise Circus	Massive Attack	9,922	triphop, electronic, downtempo
	Teardrop	Massive Attack	9,891	triphop, electronic, downtempo
	Kill Mercy within	Korn	9,484	numetal, electronic, dubstep
	Seven Nation Army	The White Stripes	9,380	rock, alternative rock, indie rock

The most popular tracks in Cluster 0 are mainly attributed to the microgenres indie rock and alternative rock. Six tracks in the top 20 have indie rock as the most associated microgenre, three alternative rock. Eight of 20 tracks have both indie rock as well as alternative rock within their five most associated microgenres. All of the 19 tracks among the top 20 that have microgenres on track level (Si Te Quisieras Venir by the Los Planetas does not have microgenres assigned on a track level), are associated with indie rock or alternative rock; most of them with both. Only a few tracks in later positions (thus, not in the top 10) deviate from these genres (e.g., Set Fire to the Rain by Adele ranks on position 14 and is associated with the genres soul and pop, Hurt by Johnny Cash is on position 16 and is mainly associated with country and folk, or Get Lucky by Daft Punk feat. Pharrell Williams on the position 20 that is associated with electronic). With 5 of the 20 most frequently listened tracks in this cluster, the band Arctic Monkeys is particularly dominant in that cluster.

While indie rock and alternative rock are represented in the most frequently listened tracks in Cluster 0 as well as Cluster 1, the tracks in Cluster 1 differentiate insofar from those in Cluster 0 as there is a tendency that the tracks include pop or electronic elements (e.g., VCR by The xx associated with electronic and indie rock or Cosmic Love by Florence + the Machine). Four tracks in the top 20 have indie pop as the most associated microgenre, 3 electronic. Ten tracks in the top 20 have indie rock as well as alternative rock as tagged microgenres. For all tracks except Hurt by Johnny Cash and Lonely Day by System of a Down, pop is one of the tagged microgenres. Electronic is associated with 9 of the 20 tracks.

In Cluster 2, two tracks that are most associated with folk are among the most popular tracks in the cluster (e.g., Little Lion Man or The Cave by Mumford & Sons). Among the top 20, there are 4 tracks associated mostly with folk. Tracks that are associated with electronic and pop (e.g., Judas by Lady Gaga) and tracks associated with triphop and electronic (e.g., Teardrop by Massive Attack) are also strongly represented. We recall Figure 10 showing that Cluster 2 has the highest average age in our sample of Last.fm users. The high average age of users in Cluster 2 and the tendency to like folk music are in line with previous research that found that folk music is more established among older users compared to younger ones (Schedl and Ferwerda, 2017; Schedl and Bauer, 2017). Yet, the results in Schedl and Ferwerda (2017) suggest that the preference for folk music is more prevalent for female than for male users; this seems not to be fully in line with the characteristics of Cluster 7 at first sight because the female/male ratio in Cluster 2 is not particularly high (Figure 12). Delving deeper on the track characteristics, though, we notice that previous works considered a rather coarse-grained taxonomy of genres, whereas the work at hand considers microgenres. Table 2 shows that the 10 most popular tracks in Cluster 2 reflect indie rock (4 out of 10), alternative rock (3 out of 10), and (indie) folk (2 out of 10). In previous work (Schedl and Ferwerda, 2017), alternative (rock) was associated rather with male users (typically with younger users, though). So the indie and alternative element may suggest a rather male audience.

While the most listened song in Cluster 3 is associated with country (It Ain’t Cool To Be Crazy About You by George Strait), this cluster shows a lot of tracks that are tagged with folk among the most popular ones for that cluster; 4 of the top 20 have it as their most associated microgenre. The folk tracks are either associated with folk and the singer/songwriter genre (e.g., Flume or Holocene by Bon Iver) or are attributed to indie folk (e.g., In the Aeroplane Over the Sea by Neutral Milk Hotel). Eleven tracks in the top 20 are associated with electronic or electronica within the track’s five most tagged microgenres.

The most popular tracks in Cluster 4 are predominantly associated with progressive rock or alternative rock (e.g., 3 Libras by A Perfect Circle). Within the top 20 of this cluster, 10 tracks are associated with some form of progressive rock and 2 with progressive metal, 14 with alternative rock, and 9 with some form of metal (i.e., progressive metal, alternative metal, doom metal, or with the gernic term metal). An interesting deviation from the dominance of the rock genre is the track World’s End by Hatsune Miku & Megurine Luka, who is a vocaloid and j-pop artist. Indeed, all playcounts for that track are generated by a single user 9 from Chile (CL); thus, this track is not representative for Cluster 4. A further deviation is constituted by Por la Ventana by Gepe associated with the genres folk and singer/songwriter, which is listened to by more than one user.

The most popular tracks in Cluster 5 are mostly associated with the psychedelic rock genre. Interestingly, 11 of the 20 most popular tracks are by the band Phase. An exception from the strong psychedelic rock representation in this cluster is the track Slow Me Down by Anneke van Giersbergen, a track that is associated with the singer/songwriter genre, while the artist is mainly associated with alternative rock and metal, but also pop-rock.

Cluster 6 is characterized by a dichotomy of genres among the most popular tracks. On the one hand, there are tracks associated with singer/songwriter and pop (e.g., If I Could and I Can’t Change by Sophie Zelmani). On the other hand, there is a strong representation of doom metal with tracks such as Without God and Day by Katatonia. Interestingly, both Sophie Zelmani as well as Katatonia are present with several songs among the most popular tracks in this cluster. Recalling Figures 10 **–** 12 that visualize the user characteristics for the eight clusters, uneven distribution with respect to the female/male ratio and the generally low playcount per user (compared to the other clusters), and the young age of its users may be characterizing aspects for Cluster 6 that result in this heterogeneous picture with singer/songwriter and pop tracks, on the one hand, and the strong representation of doom metal, on the other. For instance, Schedl and Ferwerda (2017) found pop being more popular among female than male users, while it is the opposite for metal. Interestingly, the results of Schedl and Ferwerda (2017) (considering a global sample, also relying on data from Last.fm) suggest that the age group in which the users of Cluster 7 range, is the age group that likes pop least of all analyzed age groups, and for liking of meta this age group ranges in the middle field.

The only cluster that includes many popular tracks associated with the pop genre is Cluster 7. Tracks include Skyscraper by Demi Lovato, Come and Get It by Selena Gomez, and Dark Paradise by Lana Del Rey. Next to the generic tag pop (19 occurrences), the most mentioned microgenres among the top 20 in this cluster are poprock (16 occurrences) and indie pop (13 occurrences), followed by britpop (9), electro pop (6), dance pop (6), dream pop (4), synth pop (3), chamber pop (3), alternative pop (3), teen pop (2), art pop (2), power pop (1), jangle pop (1), and k-pop (1). The high ratio of female users (Figure 12) might be a cohesive characteristic in this cluster as already previous work has shown that female users are more inclined to listen to pop music than male users, in particular in the Last.fm community (Schedl and Bauer, 2017; Schedl and Ferwerda, 2017).

Cluster 8 is characterized by the post-hardcore genre. Seven tracks in the top 20 in this cluster are tagged with post-hardhore, five of those have it as their most tagged microgenre. Triphop (8 tracks), screamo (6 tracks), and hardcore (6 tracks) are also well represented among the top 20 in this cluster. Popular tracks include Another Bottle Down by Asking Alexandria, … Meltdown by Enter Shikari, and Nineteen Fifty Eight by A Day to Remember. An interesting deviation from this post-hardcore association are, for instance, Dexter by Ricardo Villalobos (minimal techno) and Cookie Thumper! by Die Antwoord (hip hop), which are also among the most popular tracks in this cluster.

Summarizing the answer to RQ1, which we addressed here (To what extent can we identify and interpret groups of countries that constitute *music preference archetypes*, from behavioral traces of users’ music listening records?), we find nine clusters of countries, with each of the clusters representing a *music preference archetype* that reflects different nuances of music preferences in terms of the Spotify microgenres. While some *music preference archetypes* represent countries with geographical proximity (e.g., Cluster 6 and Cluster 8) and some archetypes share linguistic similarities (e.g., Cluster 3 and Cluster 8), others include interesting outliers (e.g, Iceland (IS) in Cluster 0, Israel (IL) for Cluster 4, or Brazil (BR) in Cluster 7).

### 4.2 Music Track Recommendation Using Country Context

In the following, we first detail the setup of the conducted evaluation experiments for the music track recommendation task, including evaluation protocol, baselines, and performance metrics (Section 4.2.1). Subsequently, we report and discuss the obtained results and answer the related research questions (Section 4.2.2).

#### 4.2.1 Experimental Setup

After preselection and filtering (cf. Section 3.1), the dataset contains the listening histories of 54,337 Last.fm users. To carry out the recommendation experiments, we split the data into training, validation, and test sets. For each of validation and test set, 5,000 users are randomly sampled. The original VAE model (Liang et al., 2018) and our extended VAE architecture that integrates the user context models described in Section 3.3 are trained on the full listening events of the uses in the training set. For users in the validation and test set, 80% of all listening events are randomly selected to act as an input for the model, and the remaining 20% are used for evaluation. The NDCG@100 metric (see below) on the validation set is used to select the hyperparameters of our models.

*Baselines*: In addition to comparing our extended context-aware model to the original VAE recommendation architecture (Liang et al., 2018), we also include two baselines in the experiments, i.e., variants of most popular (MP) and implicit matrix factorization (IMF). In the *most popular* (MP) models, a popularity measure is calculated for each track based on its sum of listening events across users in the training set. We implemented and evaluated three flavors of MP: *MP global* computes the most popular tracks on a global scale (independent of country); *MP country* considers only the top tracks in the country of the target user; *MP cluster* considers only the top tracks within the cluster the country of the target user belongs to. We then rank tracks accordingly and use the ranking to produce recommendations, which are evaluated on the 20% split of the test set (for each user). To make results between the baseline and our proposed model comparable, we exclude tracks that are part of a user’s known listening history, i.e., listening events from the remaining 80%. As a second baseline, we adopt a collaborative filtering approach using *implicit matrix factorization* (IMF) according to Koren et al. (2009). We use the implementation provided by Spotlight^16^ with random negative sampling (50:50), 128 latent dimensions, and a pointwise loss function.

*Performance metrics* **:** To quantify the accuracy of the recommendations, we use the following metrics (similar to Liang et al., 2018; Schedl et al., 2018; Aiolli, 2013), which we report averaged over all users (in the test set). Thus, for each user in the test set, we generate recommendations using the data in the training set and compare the recommended tracks with the actually listened tracks of the user present in the test set in order to calculate the performance metrics. Note that we use the definitions common in recommender systems research, which are partly different from the ones traditionally used in information retrieval. *Precision@K* for user *u*:

$$P@K\left(u\right)=\frac{1}{K}\overset{K}{\underset{i=1}{\sum }}rel\left(i\right),$$ (9)

where *K* is the number of recommended items and rel(i) is an indicator function signaling whether the recommended track at rank *i* is relevant to *u* or not. This means that rel(i)=1 if the recommended track at rank *i* can be found in the test set; rel(i)=0 if not. *Recall@K* for user *u*:

$$R@K\left(u\right)=\frac{1}{\text{min}\left(K,N_{u}\right)}\overset{K}{\underset{i=1}{\sum }}rel\left(i\right)$$ (10)

where $N_{u}$ is the number of items in the test set that are relevant to *u*, *K* is the size of the recommendation list, and rel(i) is the same indicator function as used for *Precision@K*. When comparing *Precision@K* and *Recall@K*, *Precision@K* can be seen as a measure of the usefulness of recommendations and *Recall@K* as a measure of the completeness of recommendations. *Normalized discounted cumulative gain@K*:

$$NDCG@K\left(u\right)=\frac{DCG@K\left(u\right)}{IDCG@K\left(u\right)}$$ (11)

where IDCG@K(u) is the ideal DCG@K for user *u*, achieved when all items relevant to *u* are ranked at the top, and DCG@K(u) is the *discounted cumulative gain* at position *k* for user *u*. It is given by:

$$DCG@K\left(u\right)=\overset{K}{\underset{i=1}{\sum }}\frac{rel\left(i\right)}{{\text{log}}_{2}\left(i+1\right)}$$ (12)

where rel(i) is the same indicator function as used for *Precision@K* and *Recall@K*. In contrast to those two performance metrics, *NDCG@K* is a ranking-based metric, which also takes the position of the recommended tracks into account since higher-ranked items are given more weight.

We compute and report all metrics for K=10 and K=100, simulating users who are just interested in a few top recommendations and users who inspect a large part of the recommendation list, respectively.

#### 4.2.2 Results and Discussion

Table 3 shows the performance achieved on the test set, averaged over all users in the test set. As a general observation, we see that the VAE-based approaches outperform the baselines (MP and IMF) by a substantial extent. Of the baselines, IMF performs superior to MP global while the other two variants of MP (MP country and MP cluster) yield better results than IMF. The poor performance of MP global is somewhat surprising since several studies (e.g., Tiwari et al., 2018; Lai et al., 2019; Vall et al., 2019) have shown that recommendation approaches leveraging popularity information—e.g., always suggesting the items that are most frequently consumed—often achieve highly competitive accuracy values in offline experiments, despite the obvious fact that such recommendations will likely not be perceived very useful by the users. A likely reason is that we perform track recommendation while the earlier mentioned works commonly adopt an artist recommendation setup. In an artist recommendation scenario, it is very likely that a user has consumed every highly popular artist at least once. This leads to a high performance of a popularity-based approach. In the track recommendation scenario adopted in the work at hand, the granularity of items (tracks vs. artists) is higher and—in comparison to the artist recommendation scenario—it is not necessarily the case that the most popular tracks have been consumed by most users at least once. Overall, a popularity-based approach may work well for artist recommendation but less so for the more fine-grained track recommendation.

**TABLE 3:** Results with respect to Precision@K, Recall@K, and NDCG@K metrics.

Model	P@10	P@100	R@10	R@100	NDCG@10	NDCG@100
MP global	0.048	0.033	0.048	0.036	0.050	0.037
MP country	0.203	0.156	0.203	0.157	0.209	0.166
MP cluster	0.193	0.149	0.193	0.149	0.199	0.158
IMF	0.080	0.072	0.080	0.064	0.081	0.071
VAE	0.482	0.309	0.486	0.367	0.500	0.383
VAE country id (model 1)	0.513	0.325	0.517	0.384	0.532	0.402
VAE cluster id (model 2)	0.515	0.326	0.520	0.385	0.537	0.404
VAE cluster dist (model 3)	0.513	0.325	0.518	0.384	0.534	0.403
VAE country dist (model 4)	0.516	0.325	0.521	0.383	0.535	0.403
VAE sampling	0.224	0.099	0.239	0.255	0.252	0.223
VAE sampling country id (model 1)	0.230	0.102	0.245	0.259	0.259	0.227
VAE sampling cluster id (model 2)	0.231	0.101	0.246	0.259	0.261	0.227
VAE sampling cluster dist (model 3)	0.232	0.102	0.245	0.258	0.246	0.258
VAE sampling country dist (model 4)	0.225	0.100	0.239	0.255	0.255	0.223

For all metrics, pairwise comparison using a Wilcoxon signed-rank test shows significant improvements from MP global to IMF to VAE to all VAE models with context (models 1–4); there are no significant differences between the 4 VAE models that use context, though. The five rows at the bottom (“VAE sampling …”) show results for another set of experiments in which we randomly sampled (three times) exactly 122,442 tracks from about 1 million tracks instead of computing performance measures on the top 122,442 tracks of the whole collection as done in the main experiment.

On the other hand, we also note that the other two variants (MP country and MP country) achieve much better results than MP global, even outperforming the IMF approach. This might be explained by the more narrow but better user-tailored consideration of the country-specific mainstream (cf. Bauer and Schedl, 2019), which is reflected in the computation of most popular tracks in the MP country and MP cluster models.

Comparing the proposed context-aware extensions of the VAE recommendation architecture to the original VAE (Liang et al., 2018), we observe a clear improvement of all metrics when integrating the user context models. This improvement is achieved irrespective of the actual user model we adopt (models 1–4). Precision@10 increases by 3.4 percentage points (7.1%) from VAE to the best performing VAE context model (model 4) that leverages the distances between users and country centroids. Likewise, Precision@100 increases by 1.7 percentage points (5.5%). Recall@10 and Recall@100 improve, respectively, by a maximum of 3.5 percentage points (7.2%), realized by model 4, and by 1.8 percentage points (4.9%), realized by model 2. In terms of NDCG, the largest gains are realized by VAE context model 2 that incorporates cluster ids. NDCG@10 improves by 3.7 percentage points (7.4%) compared to VAE; NDCG@100 increases by 2.1 percentage points (5.5%).

We investigate statistical significance of the results as follows. For all used metrics (i.e., P@10, P@100, R@10, R@100, NDCG@10, NDCG@100), data is non-normally distributed (Kolmogorov-Smirnov test, p≤0.001). Accordingly, we use the Friedman test (Friedman, 1937) to test the models’ performances for differences. For each metric, the models differ at a significance level of p≤0.001. In pairwise comparisons using a Wilcoxon signed-rank test (Wilcoxon, 1945), for each metric, the tests indicate that VAE outperforms each of the baselines (i.e, MP an IMF) at a significance level of p≤0.05. Furthermore, we perform a pairwise comparison, again using Wilcoxon’s signed-rank test, for each metric and each pair of pure VAE and one of the models integrating context information (i.e., models 1–4). For each metric and each of the models 1–4, the models 1–4 outperform the pure VAE (without context integration) at a significance level of p≤0.05. Yet, the Friedman test did not indicate any significant differences of the models 1–4 for any of the metrics.

Returning to the original research questions, we answer RQ2 (Which are effective ways to model the users’ geographic background as a contextual factor for music recommendation?) by pointing to the fact that all four user models proposed are effective to significantly improve recommendation quality in terms of precision, recall, and NDCG measures. We note, however, that performance differences between the four user context models are largely negligible. In summary, leveraging country information for music track recommendation (either as country or cluster identifier, or as distances between the target user and each cluster’s centroid) is beneficial compared to not including any country information.

As for RQ3 (How can we extend a state-of-the-art recommendation algorithm to include user context information, in particular, our geo-aware user models?), we proposed an extension of a state-of-the-art recommender based on a VAE architecture (Liang et al., 2018), i.e., we devised a multi-layer generative model in which contextual features can influence recommendations through a gating mechanism.

To investigate the generalizability of our findings to a dataset with different characteristics, we perform an additional experiment as follows. We estimate performance on a more diverse dataset in terms of track popularity than the one that considers only the top 122,442 tracks. More precisely, we create a second dataset by first considering all tracks that have been listened to as least 100 (instead of 1,000) times, yielding 1,012,961 unique tracks. We then randomly sample, three times, exactly the same amount of tracks (122,442) as used in our main experiment, and we evaluate the VAE approaches on each randomly sampled subset, averaging performance measures across the three runs.^17^ Results can be found in the five last rows of Table 3 (models named “VAE sampling …”). While we observe an obvious decrease in performance when considering items further down the popularity scale, results are still in line with the findings obtained on the main dataset. In particular, our extended VAE models (models 1–4) still outperform the original VAE architecture, with respect to all performance metrics.

## 5 Conclusions, Limitations, and Future Work

In summary, we approached the task of identifying country clusters and corresponding archetypes of music consumption preferences based on behavioral data of music listening that originates from Last.fm users. Together with the users’ self-disclosed country information, we used the listening data (369 million listening events created by 54 thousand Last.fm users) as an input to unsupervized learning techniques (t-SNE and OPTICS), allowing us to *identify nine archetypal country clusters*. We discussed these clusters in detail with respect to their corresponding users’ music preferences on the track level and the linguistic, historical, and cultural backgrounds of the countries in each cluster. Additionally, we considered the distribution of age, gender, and average playcount per user as aspects in our analysis.

Furthermore, we proposed a context-aware music recommendation approach operating on the music track level, which integrates different user models that are based on the user’s country or country cluster. To this end, we *extended a variational autoencoder (VAE) architecture by a gating mechanism to add contextual user features*. We considered *four user models*, either encoding the target user’s country information (model 1) or cluster information (model 2) directly, or as a feature vector containing the distances between the target user and all cluster centroids (model 3) or all individual country centroids (model 4). In evaluation experiments, using precision, recall, and NDCG as performance metrics, we showed that all VAE architectures outperformed a popularity-based recommender and implicit matrix factorization, which served as our baselines. Results further revealed *superior performance of all VAE variants that include context information* vis-à-vis VAE without context information, regardless of how country information is encoded in the user model.

Yet, this work has potential **limitations** with respect to the underlying dataset, which we discuss in the following. There are social patterns that define how and why people access music (López-Sintas et al., 2014). A dataset containing logs of the interactions with an online platform can, thus, only capture those listening events of people using any form of online music platform. According to López-Sintas et al. (2014), music access patterns are structured by an individual’s social position (indicated by education) and life stage (indicated by age). A bias with respect to the users’ social background can therefore be expected for our dataset. For instance, the dataset has a strong *community bias* toward users in the United States (US), while other countries are less represented. Furthermore, *user information is self-reported* by the users, which may be prone to errors and may not necessarily reflect the truth. For instance, some users report as their country Antarctica (AQ) or a birth year of 1900, which both do not seem overly plausible—especially in combination (also see Figure 1 in Schedl, 2017). Moreover, some users show *very high playcounts for single tracks*, which are not popular among other users. This also affects six of the tracks presented in our discussion of the music preference archetypes. For instance, World’s End by Hatsune Miku & Megurine Luka has a playcount of 1,228 generated by a single unique user. Similarly, One Thing’ by Runrig and Resemnare by Valeriu Sterian both have exactly one unique listener, who generated a playcount of 4,000 and 3,591, respectively. The track Ariane by Nova has 3 unique users; I Can’t Change [New Song] and To Know You (Alt. Version)—both by Sophie Zelmani—have 5 unique users each, whereof almost all playcounts were generated by only one single user. For both songs, this is the same user. Notably, also the preferences of the Last.fm users in our dataset toward certain genres differ from the genre preferences of the population at large. For instance, we found that rap and R&B as well as classical music is substantially underrepresented in Last.fm listening data (Schedl and Tkalcic, 2014), which we use in the present study. To some extent, these limitations related to the dataset could be alleviated in the future by performing further data cleansing and preprocessing steps, e.g., threshold-based filtering of exorbitant playcounts by a minority of listeners.

Another limitation of the work concerns a characteristic of t-SNE, which is that the *cost function t-SNE uses is non-convex*. This, in turn, may result in a different embedding of data points in the low-dimensional output space when the t-SNE algorithm is run on different software or hardware configurations.^18^ Please note that this does not only concern the present work, but potentially the entire (large) body of research that employs t-SNE for visualization. It is, however, an aspect that is barely discussed. We address this issue in the current work by providing exact details on our implementation and used software, and by releasing to the public the source code, parameter configurations, and dataset used in our experiments.

In this work, we used simple mechanisms to integrate country information as context factors into a VAE architecture. While they worked out well, i.e., outperformed a non-context-aware VAE, we expect even better performance with other user models, whose creation will be part of **future research**. For instance, we contemplate using *probabilistic models* to describe the likelihood of each user to belong to each cluster (or country), e.g., via Gaussian mixture models. Given the actual country of a user, we could then analyze in more detail users whose stated country is not the country with highest probability. Such a framework could also be used to *diversify recommendations* according to a user-selected country, fulfilling user intents such as “I want music of my preferred genre, but listened to by Brazilians”.

Furthermore, it would be worthwhile to *compare the clustering and recommendation results* we achieved here on the track level to results achieved when modeling music preferences on the artist level, keeping all other methodological details the same. In particular, since previous studies have predominantly shown that popularity-based music recommendation systems perform well when recommending artists, such a comparison could be enlightening.

Finally, we aim at delving into the possible *cultural, historical, or socio-economic reasons* that may underlie the differences in music preferences between the identified archetypes. To this end, we will consider theories and insights from cultural sciences, history, sociology, and economics, and connect our music preference archetypes to these theories. Another promising path for further analysis of the country clusters is to consider dimensions rooted in the music market or the music content itself, including considerations such as local demand, production of music styles, reception of music styles, diffusion, etc., as well as dimensions related to the users’ listening habits.

## Data Availability

To foster reproducibility, we release the code and data used in this work to the public. The code can be found on https://gitlab.cp.jku.at/markus/fiai2020_country_clusters; the dataset (Schedl et al., 2020) is available from https://zenodo.org/record/3907362#.XvRq1CgzZPY. In our experiments, we used the following setup: Windows 10 Home, Build 18,362; Python 3.7.0; T-SNE and OPTICS implementations of scikit-learn 0.21.3. The remaining, system-independent, configuration details can be found in the code.

## Footnotes

1
https://geerthofstede.com/research-and-vsm/dimension-data-matrix

2
https://qog.pol.gu.se

3
https://worldhappiness.report

4
http://www.cp.jku.at/datasets/LFM-1b

5 The LFM-1b dataset used in our study is considered derivative work according to paragraph 4.1 of Last.fm’s API Terms of Service (https://www.last.fm/api/tos). The Last.fm Terms of Service further grant us a license to use this data (according to paragraph 4).

6 Please note that country-specific results may still be influenced by some users showing particularly high playcounts. Nevertheless, we decided against excluding or penalizing the listening information of such users just because users with a high playcount indicate a more pronounced inclination to listen to music. Our reasoning is that users who contribute only few listening events to Last.fm should be considered less important to model their country-specific listening behavior than users who heavily contribute. In addition, removing such “power listeners” would distort the original distribution of usersâ€™ playcounts in the sample.

7 Reducing the dimensionality of the dataset to 50 dimensions preserves only 90.1% of the variance.

8 In this work, we use Euclidean distance as distance metric and set ξ=0.05.

9 More precisely, we performed grid search on t-SNE perplexity in the range [1, 2, 3, 5, 10, 15, 20, 25, 30, 35, 40, 50] and on the minimum number of data points per cluster enforced by OPTICS in the range [2, 3, 4, 5], optimizing for average neighborhood preservation ratio (nearest neighbor consistency).

10
https://www.iso.org/iso-3166-country-codes.html

11
https://www.last.fm/api/show/track.getTopTags

12
http://everynoise.com

13 We also run experiments in which we simply concatenate track listening history and context information, but this did not show improvements over the VAE based on just the listening history.

14 Please note that observations concerning age relate to our sample of Last.fm users.

15 We released the full list of the top 20 tracks (and corresponding artists) per cluster and all microgenre annotations (for each track and artist).

16
https://maciejkula.github.io/spotlight/factorization/implicit.html

17 Please note that computational limitations prevented us from running experiments on all 1,012,961 tracks, even more so on the entire LFM-1b dataset.

18 Note that our results are stable for a given machine, software configuration, and parameter setting since we fixed the seed of the random number generator. Running the code on other configurations, however, may result in a slightly different visualization and clustering.
